# Supplementary material for: Isogenic GAA-KO Murine Muscle Cell Lines Mimicking Severe Pompe Mutations as Preclinical Models for the Screening of Potential Gene Therapy Strategies
Source: Int J Mol Sci. 2022 Jun 4;23(11):6298. doi: 10.3390/ijms23116298 (PMC9181599; doi:10.3390/ijms23116298)
Supplement: Supplementary file 1 [file ijms-23-06298-s001.zip › ijms-1741098-supplementary.pdf]

Supplemental Materials

Isogenic GAA-KO Murine Muscle Cell Lines Mimicking Severe Pompe Mutations as Preclinical Models for the Screening of Potential Gene Therapy Strategies

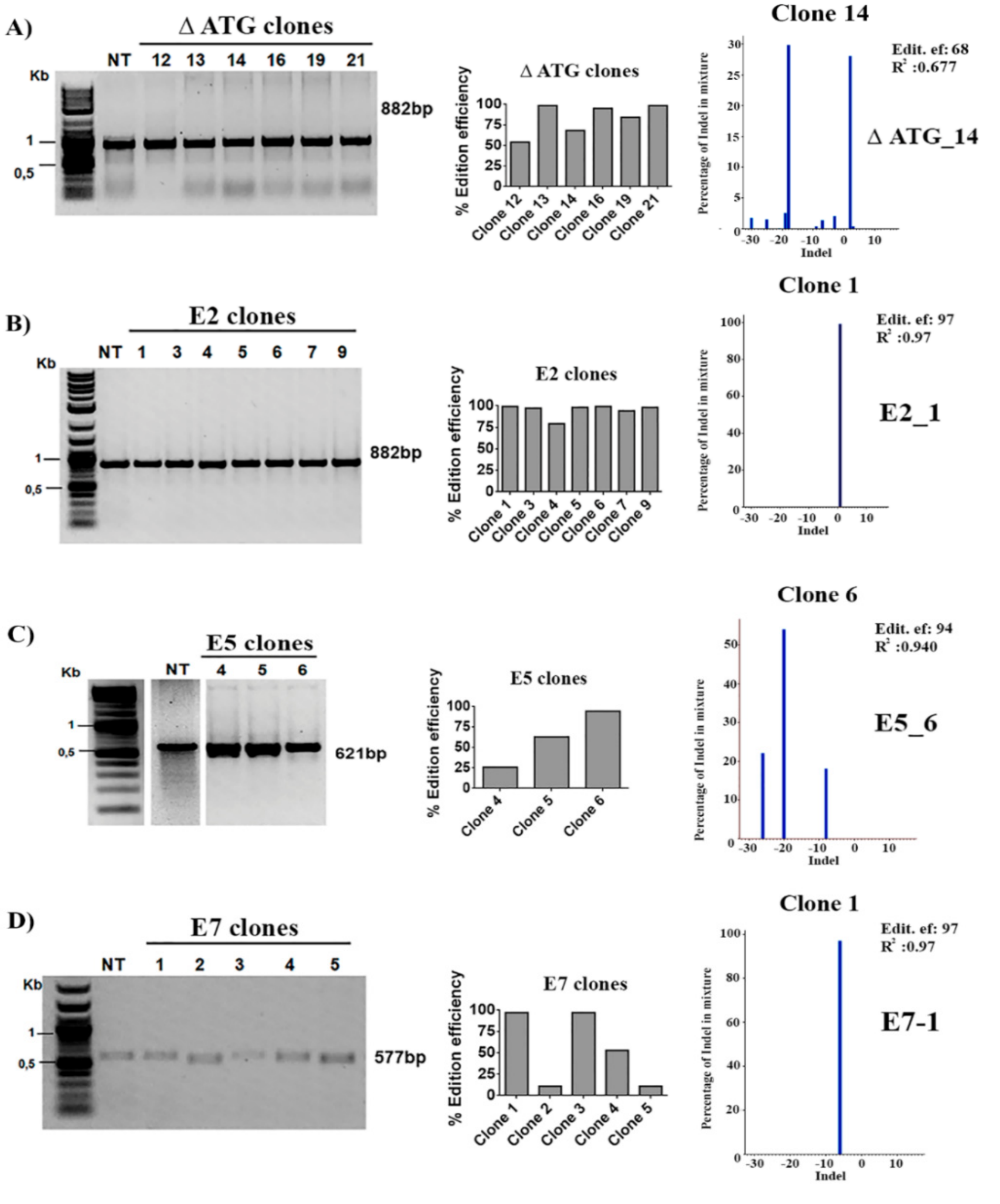

Figure S1. Analysis of different clones from Sol8 cells nucleofected with Cas9/RNAg RNPs for the generation of different mutations homologous to those found in Pompe patients; Δ ATG, E2, E5 and E7. Agarose gel showing the PCR of the amplified fragment (right panel) that was sequenced in order to determine the efficacy (middle graph) of several clones obtained from Sol8 cells nucleofected with Cas9/RNAg RNPs harbouring the sgRNA_ Δ ATG; GAGGGGCTTCCGTATATTCA (A, Δ ATG clones), the sgRNA_E2; ATCTCACAGGAGCAATGCG (B, E2 clones), the sgRNA_E5;

TTGCTAAACAGCAATGCCAT (C, E5 clones), and the sgRNA_E7; AGGTAGTGGA-GAACATGACC (D, E7 clones). The graphs showing the indels of the selected clones for each type of mutation (Δ ATG_14, E2_1, E5_6 and E7_1) are shown at the right. In this graph, the co-ordinate 0 represents the cut site, negative values represent deletions of different length and positive values represent insertions of different lengths.

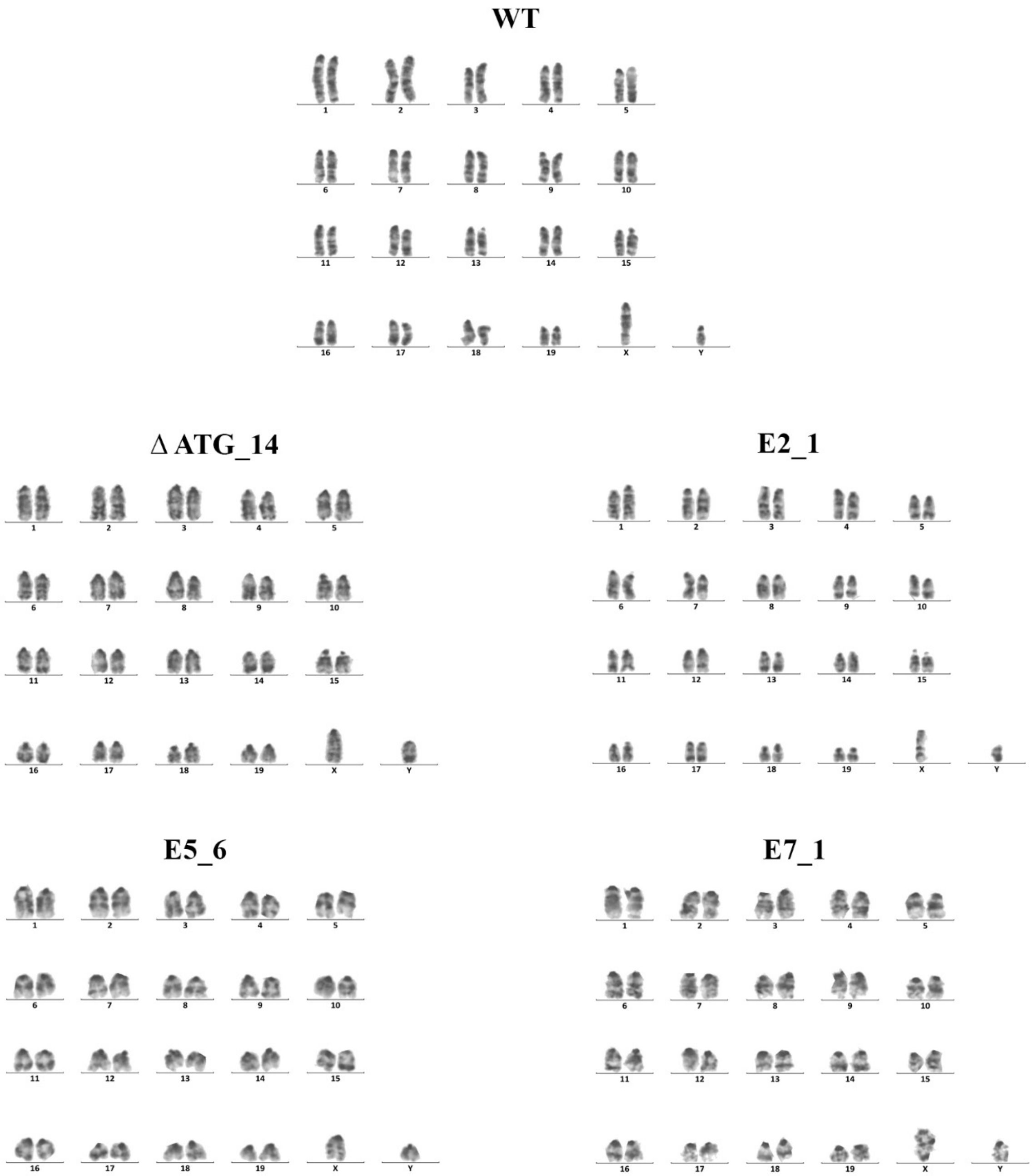

Figure S2. Cytogenetic analysis of Sol8 cells (WT and generated clones: Δ ATG_14, E2_1, E5_6 and E7_1). Normal chromosome set in all murine cell lines generated. Karyotype 40, XY (G-band technique).

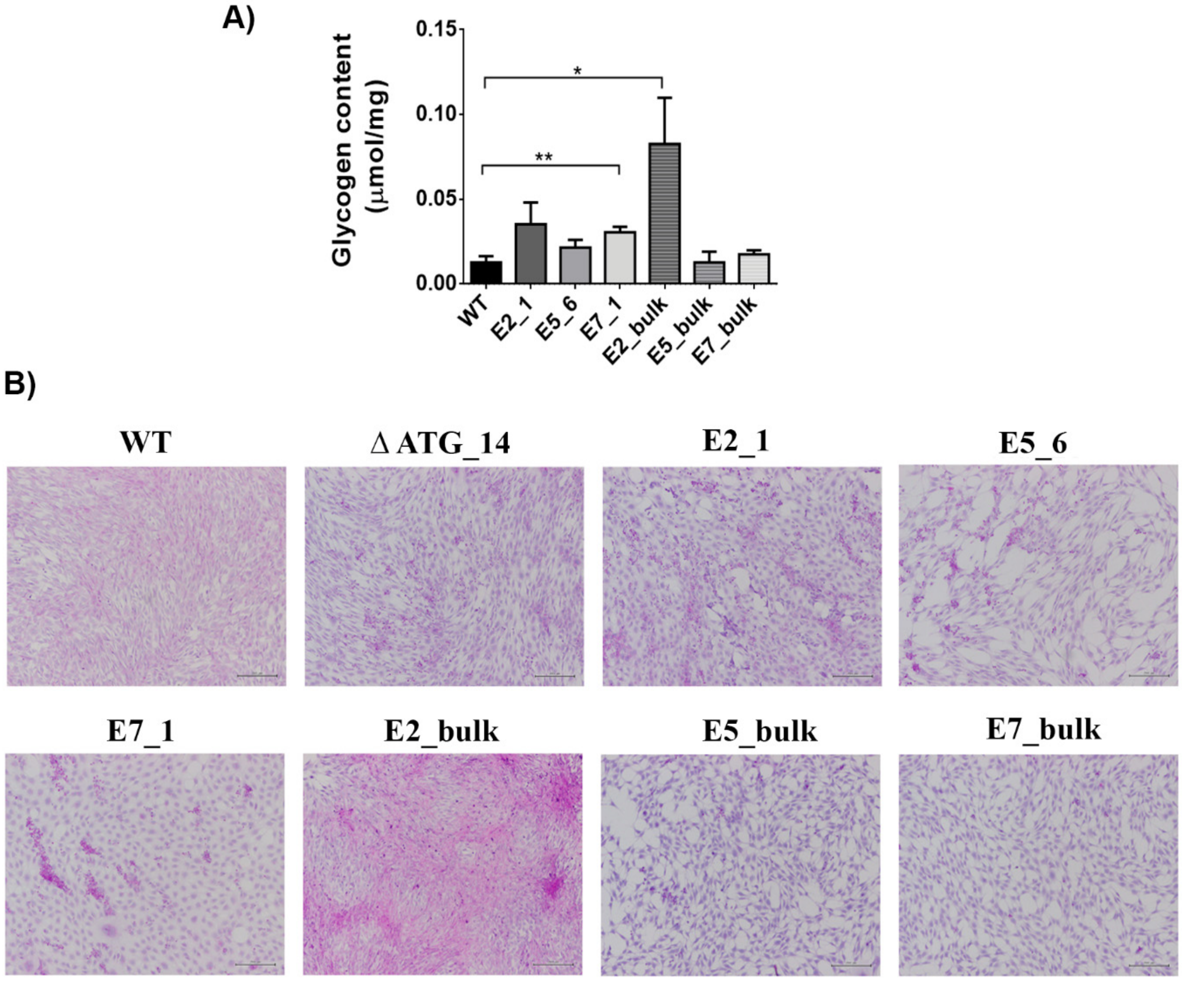

Figure S3. Glycogen accumulation in GAA-KO Sol8 clones differentiated into myotubes. A) Glycogen content in Sol8 cell myoblasts differentiated into myotubes (see M&M) in clones (E2_1, E5_6 and E7_1) and bulk populations (E2_bulk, E5_bulk and E7_bulk) compared to Sol8 wild-type (WT). B) PAS staining for glycogen visualisation in GAA-KO Sol8 clones and bulk population after differentiation into myotubes. Images obtained with an Olympus upright BX43 microscope (10x objective). Representative images are shown. Scale bar 200 μm.

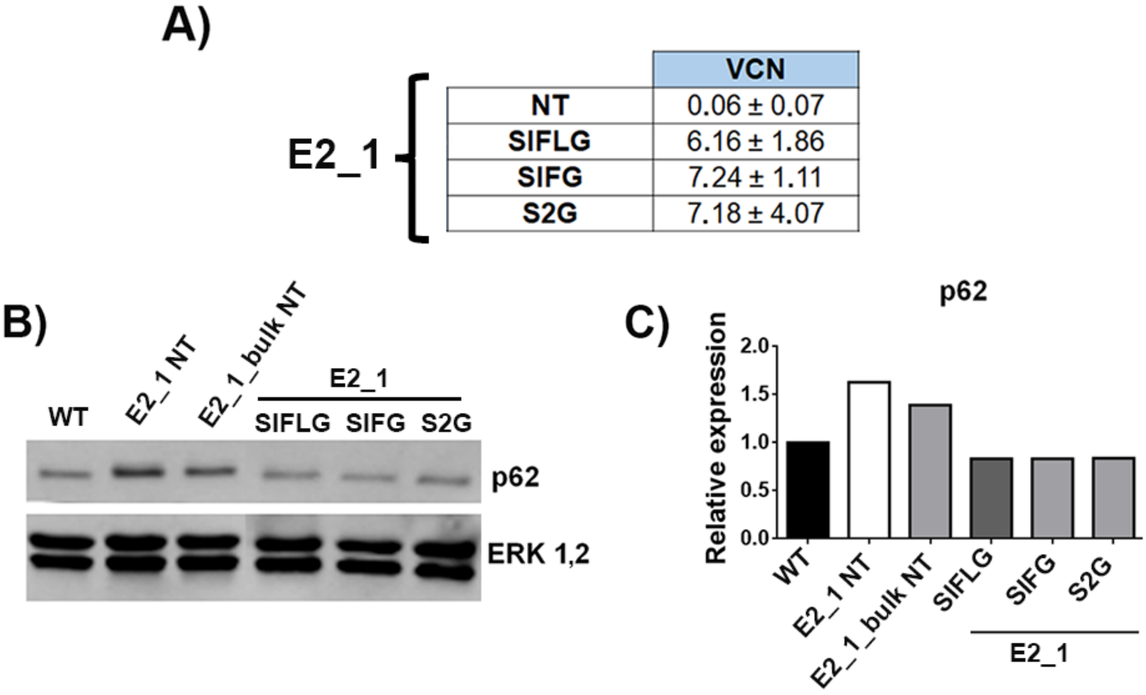

Figure S4. Rescue of p62 levels in Sol8 E2_1 cells transduced with LVs expressing the different mGAA chimeras. A) Vector copy number (VCN) of murine cells Sol8 E2_1 transduced with lentiviral vectors (LVs) expressing the different murine GAAs chimeras, SIFLG, SIFG and S2G. Western-blot images of p62 (B, top) and ERK1,2 (B, bottom) and quantification (C) of p62 expression levels in Sol8 wild-type (WT), E2_1 clone (E2_1 NT), E2_bulk (E2_bulk NT) and the E2_1 clone transduced with the different LVs (SIFLG, SIFG and S2G). The quantification was normalised to ERK1,2 levels using the ImageJ program. https://imagej.nih.gov/ij/.

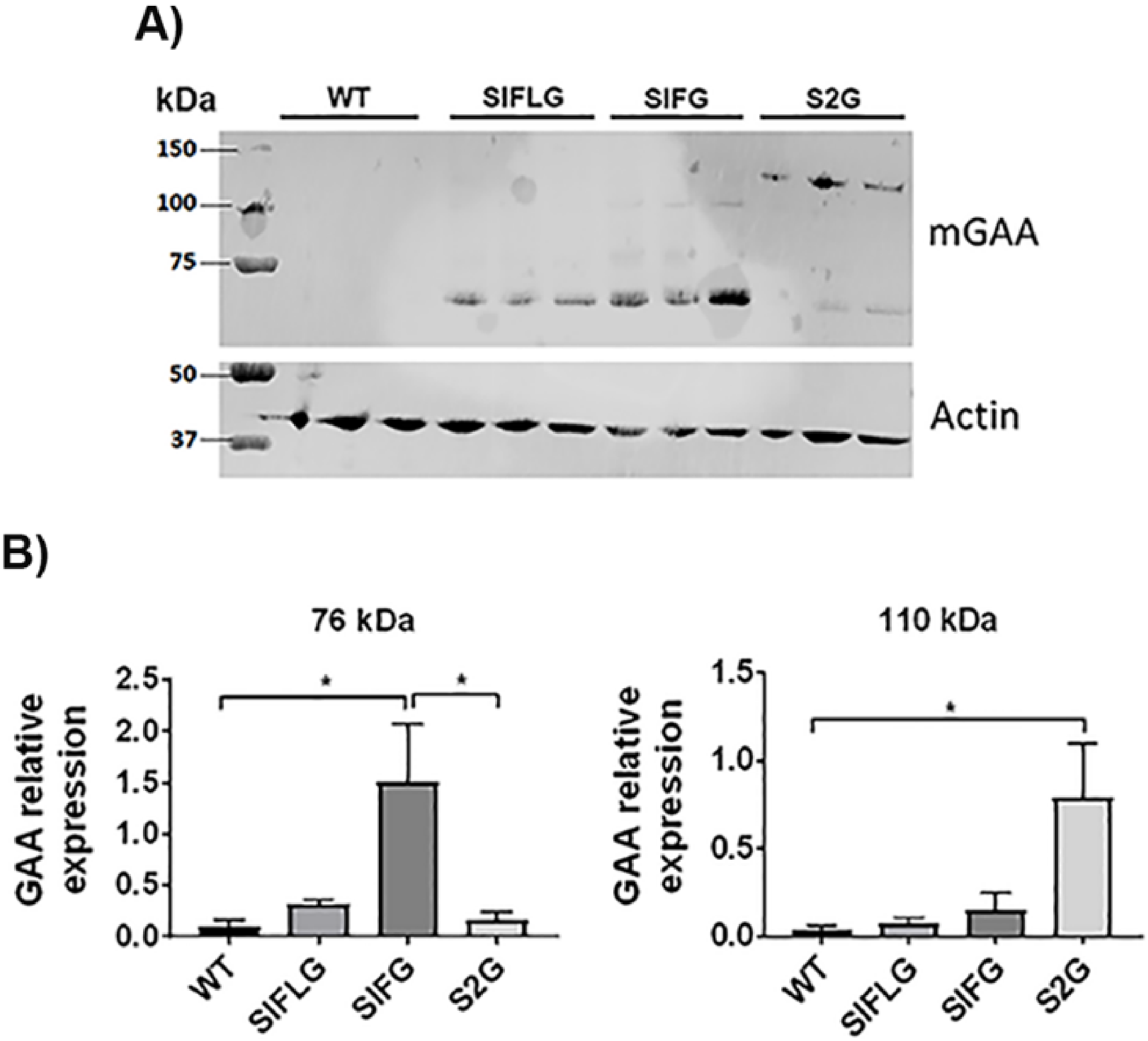

Figure S5. Intracellular GAA isoforms in Hepa 1-6 transduced cells. Western-blot image of GAA (A, top) and actin (A, bottom) expression levels in Hepa 1-6 cells (a murine model of hepatocellular carcinoma) (WT) and Hepa 1-6 transduced with the different LVs (SIFLG, SIFG and S2G) (triplicates). B) Quantification of intracellular processed (76 kDa) and unprocessed (110 kDa) mGAA levels normalised to actin levels. Quantification was performed using the ImageJ program. https://imagej.nih.gov/ij/. Statistical analyses were performed with unpaired t-test (two tails, *$p<0.05$). Values represent means +/- SEM.

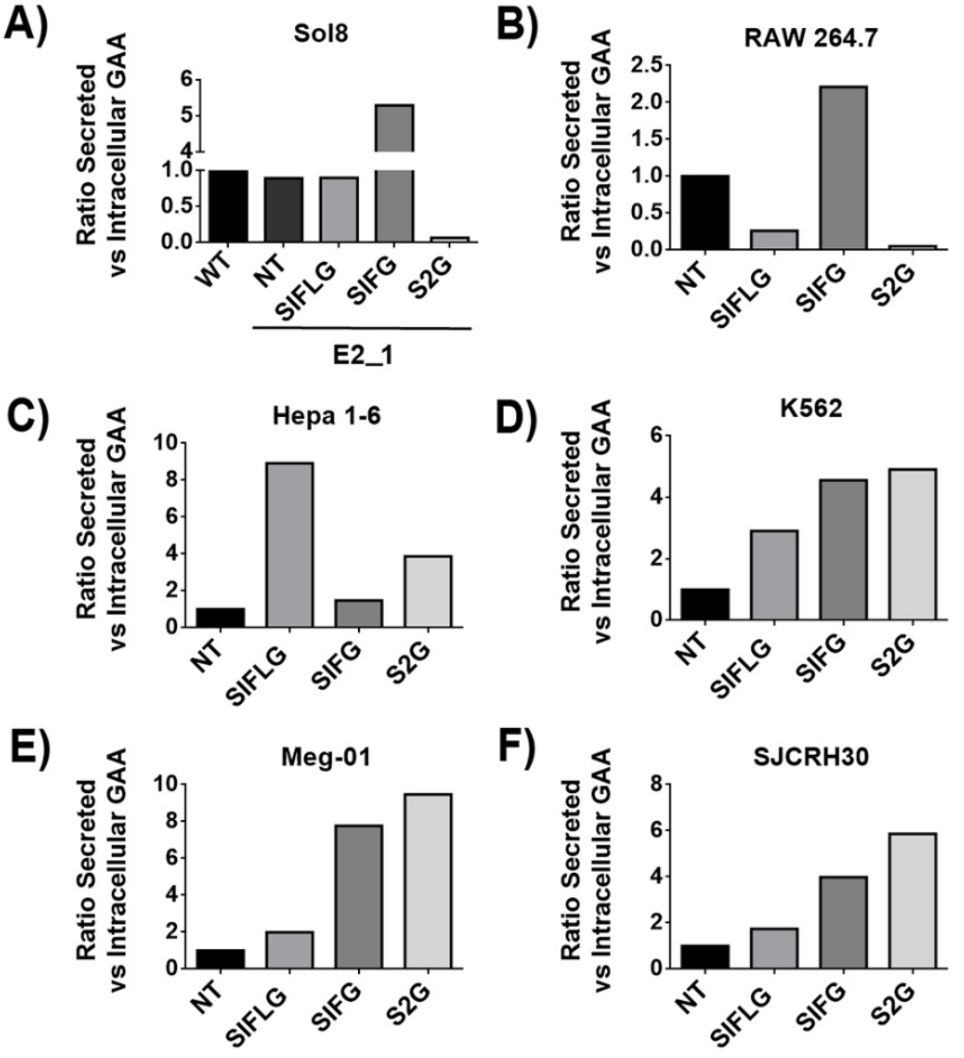

Figure S6. Secreted GAA vs. intracellular GAA ratio in E2_1 transduced cells, RAW 264.7, Hepa 1-6, K562, Meg-01 and SJCRH30 cells. Analysis of secreted versus intracellular GAA analysed by Western-blot in Figures 4B,E,F and Figure 5. Quantification was performed using the ImageJ program. https://imagej.nih.gov/ij/.

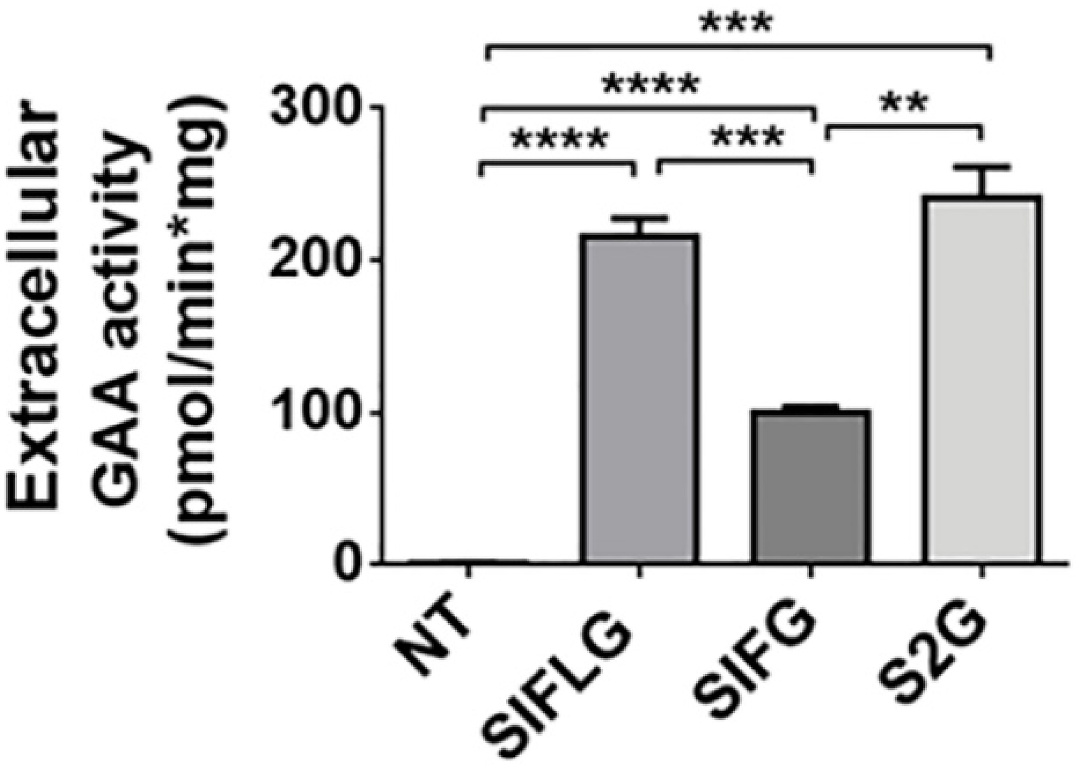

Figure S7. Activity of the GAA secreted to the media by transduced K562. Analysis of mGAA activity in the media secreted by transduced K562 cells (conditioned media) with the different constructs. NT cells were used as negative control (non-transduced K562 cells). Unpaired t test (two tails, *p<0.05). Values represent means +/- SEM of at least three separate experiments (*p<0.05).

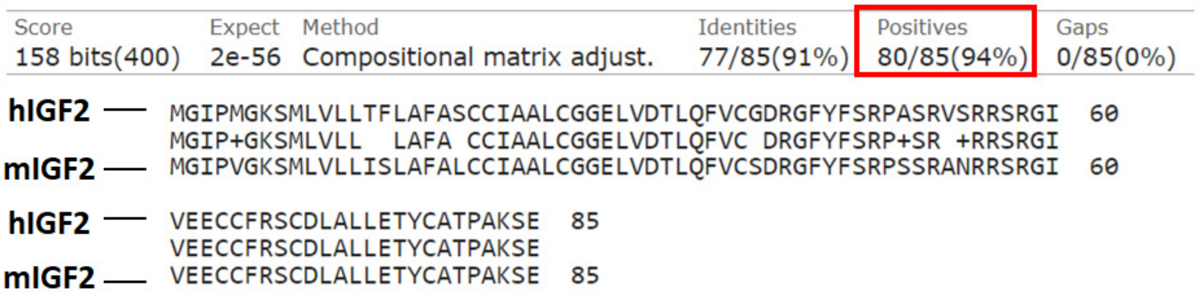

Figure S8. Comparison of IGF2 sequences. Blast analysis of the mice and human IGF2 amino acid sequences used in the murine IGF2-GAA (2G) and the human IGF2-GAA chimeras.

Table S1. Comparison of GAA mutations in Sol8 clones and those found in similar regions in Pompe patients. The mutations generated in the different GAA-KO murine muscle cell models (Δ ATG_14, E2_1, E5_6 and E7_1) were obtained by sequencing their GAA cDNA. The location of the mutation (location), the affected alleles (Allele), type of mutation (DNA nomenclature) and type of protein mutation (Protein nomenclature) are shown for each Sol8 clone (left, GAA-KO murine muscle cell models) and for Pompe patients harbouring equivalent mutations. Clones E2_1 and E7_1 are homozygous for c.306_307insT and c.1176_1181del mutations, respectively. Clones Δ ATG_14 and E5_6 have a different mutation in each allele (as described in the table). Pompe patients' mutations were obtained from Pompe variant database (https://www.pompevariantdatabase.nl). Similar mutations from Pompe patients that mimic what occurs in our generated GAA-KO models were selected.

	**GAA-KO murine muscle cell models**				**Pompe patients' mutations from database of the Pompe Center**				
	**Location**	**Allele**	**DNA nomenclature**	**Protein nomenclature**	**Location**	**DNA nomenclature**	**Protein nomenclature**	**Predicted severity**	**Phenotype with null allele**
**Δ ATG_14**	Exon 2	1	**c.-11_8del**	p.(0)	Exon 2	**c.3G>A**	p.(0)	Very severe	Classic infantile or childhood
		2	**c.4_5insT**	p.(Asn2Ilefr*43)	Exon 2	**c.18_25del**	p.(Cys8Profs*24)	Very severe	Classic infantile
**E2_1**	Exon 2	1,2	**c.306_307insT**	p.(Lys103Cysfr*85)	Exon 2	**c.340_341insT**	p.(Lys114Ilefs*32)	Very severe	Classic infantile
**E5_6**	Exon 5	1	**c.950_957del**	p.(Met317Hisfs*83)	Exon 6	**c.982_988del**	p.(Leu328Glyfs*62)	Very severe	Classic infantile
		2	**c.943_962del**	p.(Ser314Thrfs*82)					
**E7_1**	Exon 7	1,2	**c.1176_1181del**	p.(Arg393Thr394del)	Exon 8	**c.1199_1210del**	p.(Val400Asn403del)	Very severe	Classic infantile

Table S2. Off-target selection and primers of Sol8 GAA-KO clones. Description of potential off-targets selected from Synthego CRISPR design tool for the different gRNAs (g1, gCys, gE5 and gE7) used for the generation of Δ ATG_14, E2_1, E5_6 and E7_1 clones. Primers selected for their analysis are shown (right). A PCR for each off-target was performed, PCR product sequenced and an ICE analysis. No indels were found (no off-target could be detected).

	**Mismatches**	**Potential off-targets**	**Primers**	
**g1 (score = 81)**	3	GAGGGGCTTCCGTGGAATCA	Fw g-ATG OFF1	AGTGACCTCCTCCAAGTTTC
		chr15	Rev g-ATG OFF1	GTTTCATAGGAACAGCATCTTC
	3	GAGGGGCTTCTGTTTATTCT	Fw g-ATG OFF2	CAGACTCATCAGCCAACTTG
		chr8	Rev g-ATG OFF2	TGATGTCCCAGCTCCTTG
	3	GAGGGGCTTCCTTAATTTCA	Fw g-ATG OFF3	CTGCCTCAAATTCTCTTGG
		chr11	Rev g-ATG OFF3	GCTAAGCTACTTCCCTGTCAA
	3	GAGGGGCTTCCCAATATCCA	Fw g-ATG OFF4	CAACAGAACTCTAGAGCCCC
		chr17	Rev g-ATG OFF4	CCATCTCTCCAGCAATTCTTA
	4	TGGTGGCCTCCGTATATTCA	Fw g-ATG OFF6	GACTTCCCTAAGCCTGCTCTT
		chr3	Rev g-ATG OFF6	AGTGGGATTGGCCAGTGAGT

	4	GAGGAGCTTACGTATAACCA	Fw g-ATG OFF7	AGCAAGGAACCCCTGAAGATG
		chr2	Rev g-ATG OFF7	TCATGTCTGTGTGCACGGTT
	4	TAGGGGCTTACGTGTTTTCA	Fw g-ATG OFF8	AGGCCAACGTGTAAGTGCTA
		chr18	Rev g-ATG OFF8	CAGCTCCACTCACTTCCCAC
	4	GAAGGGTTTCTGTAAATTCA	Fw g-ATG OFF9	CAGAGCCTGGCACGAATATG
		chr6	Rev g-ATG OFF9	TCCTTGGCCAGAATACCTCCT
	4	GAGGAGCTTCAGAATTTTCA	Fw g-ATG OFF10	TTGGTGCAGGATAATCAGGTGG
		chr10	Rev g-ATG OFF10	AGAGGTTCTGGGTTCAGGTG
**gCys (score = 55)**	3	CATCTCACAGCAGAAATGCA	Fw g- Cys OFF1	CCCCAGGTTACCTATGGAGGA
		chr13	Rev g- Cys OFF1	AGCCTATCTACGTTTGCCCC
	3	CATCTCAAAAGAGCAATGCT	Fw g- Cys OFF2	CTTCGGCTTCAGACAGTCCTT
		chr8	Rev g- Cys OFF2	GCAACTTCAAGTGTCACATCC
	3	CATCTCACAGGAGTAATGGA	Fw g- Cys OFF3	ACAGATCCCATGACCTTGCTC
		chr16	Rev g- Cys OFF3	AGTAATGCACCCTGGAGAAGC
	3	CATCTCACAAGAGGAATGCT	Fw g- Cys OFF4	CCAGTGCACTTCCTCCCATT
		chr9	Rev g- Cys OFF4	CAAGTGGCTGTCCCGAATCT
	3	GATATCACAGGAGCAGTGCG	Fw g- Cys OFF5	GTCAAACGCAGGTGACATCC
		chr5	Rev g- Cys OFF5	TGTGTGTGTGCTAGGAACCG
	3	CATCTCACCAGAGCAATGCT	Fw g- Cys OFF6	AGCCTTGACATTTTCGAATGGT
		chr15	Rev g- Cys OFF6	TGAGGTCTGAGGACTTTTGGG
	3	CTTCTCAGAGGAGCAATGGG	Fw g- Cys OFF8	ATGACGTGAGAGTGGTGGTG
		chr1	Rev g- Cys OFF8	TCCAGTCCATTTCTGGGCAC
	3	CAGCTCACAGGAGCCATGAG	Fw g- Cys OFF9	CCTCCCCACACATCCTACCA
		chr9	Rev g- Cys OFF9	CTTCTCCACCCAGCACTCATT
**gE5 (score = 54)**	3	TTTCTAAACAGCAGTGCCCT	Fw g-MutE5 OFF1	ATCCAATTCGGTGCTGCTGT
		chr9	Rev g-MutE5 OFF1	TTGAAGAGCCAGTATGCGTGT
	3	CTGATGAACAGCAATGCCAT	Fw g-MutE5 OFF2	GCCTAGGCTGCCCTAAAAGC
		chr6	Rev g-MutE5 OFF2	TGTTGCATGCAGGAGTCTACA
	3	CTGTTGAACAGCAATGCCAT	Fw g-MutE5 OFF7	AGAAGCCAAGGTCAAACAAGG
		chr6	Rev g-MutE5 OFF7	TTGTGGTGGATTCCATGTGC
	3	ATGCTACACAGCACTGCCAT	Fw g-MutE5 OFF8	CTTGTCCTGTGGCAAGTCGT
		chr10	Rev g-MutE5 OFF8	AGGGATTGCCCGTTCGATTT
	3	TTGCTAAACATGAATGCCAA	Fw g-MutE5 OFF9	CATGAAGGAAACCGTTGAGCA
		chr6	Rev g-MutE5 OFF9	CCATGGTTTGTTTCGTGGCA

	3	TTCCTAAACAGCAATGCACT	Fw g-MutE5 OFF10	TAGGGACTGGTGAGCACTGA
		chr1	Rev g-MutE5 OFF10	GGAAGAGTCACCTCTGAGCG
**gE7 (score = 18)**	3	AGGTAGAGGAGTGCATGACC	Fw g-MutE7 OFF3	GCTGCTAGAAGCTCCACTGT
		chr17	Rev g-MutE7 OFF3	AGCACCAACGACAGTCCAAA
	3	AGGGAGAGGAGAACATGATC	Fw g-MutE7 OFF6	TCAACAGGGAAGCATGTGGT
		chr12	Rev g-MutE7 OFF6	TGGAAGTGGAGCTGGAGAGA
	3	AGGCAGTGGAGAAAAGGACC	Fw g-MutE7 OFF7	GGGTTTGGGATCCTCTGCAA
		chr11	Rev g-MutE7 OFF7	GGTGAGCACTGCCTTGATCT
	3	GGGTAGTGGAGACCAAGACC	Fw g-MutE7 OFF10	ACGTCTTCCCTGTCCGTTTC
		chr13	Rev g-MutE7 OFF10	TCCAGGGTTGCCTGTTGTTT

Table S3. Vector copy number of transduced cells. Vector copy number (VCN) of murine cells (Sol8 E2_1, RAW 264.7 and Hepa 1-6) and human cells (K562, Meg-01 and SJCRH30) transduced with lentiviral vectors expressing murine GAA (SIFLG, SIFG and S2G). Calculated by qPCR (see M&M).

		VCN
**E2_1**	**NT**	0.01 ± 0.01
	**SIFLG**	3.14 ±1.18
	**SIFG**	2.22 ±0.33
	**S2G**	3.28 ± 1.06
**RAW 264.7**	**NT**	0.01 ± 0.00
	**SIFLG**	4.11 ± 0.88
	**SIFG**	6.77 ± 2.09
	**S2G**	4.28 ± 0.46
**Hepa 1-6**	**NT**	0.01 ± 0.01
	**SIFLG**	22.19 ± 3.25
	**SIFG**	8.5 ± 1.52
	**S2G**	27.4 ± 4.66
**K562**	**NT**	0.01 ± 0.00
	**SIFLG**	14.48 ± 1.73
	**SIFG**	15.33 ± 4.65
	**S2G**	15.45 ± 4.45
**Meg-01**	**NT**	0.00 ± 0.00
	**SIFLG**	13.98 ± 5.44
	**SIFG**	34.10 ± 4.00
	**S2G**	18.10 ± 5.26
**SJCRH30**	**NT**	0.01 ± 0.00
	**SIFLG**	17.17 ± 1.63
	**SIFG**	24.57 ± 3.59
	**S2G**	17.38± 5.34
